# The Pains of Tabes Dorsalis

**Published:** 1905-03-04

**Authors:** 


### THE PAINS OF TABES DORSALIS.

In a lecture on this subject Sir Wm. Gowers draws attention to the fact that pains are far more common than the inco-ordination which prompted the name locomotor ataxia; they are also usually earlier events than ataxia, and they sometimes render life almost unendurable in cases in which there is no other subjective symptom. In other instances they are slight, but this is the exception. The less severe pains are not infrequently mistaken for rheumatism, and may be so described by the patient. Unhappily in most cases the extreme severity of the pains indicates their true interpretation and wears down the patient both by the actual suffering and by the dread of its return. A rigid classification of the varieties of pain in tabes is not possible, but the following forms may be defined. In the first class are brief, momentary attacks, succeeding each other, after a short interval, in the same place, and recurring over a period of hours or even days. Such pains may be referred either to the surface or to deeper parts. The superficial pains are most frequent at some limited area on one or other limb, but they may be experienced over the head or face; they are sharp and stabbing in character, and are responsible for the term "lightning" pains. One fact about these brief superficial pains is that they make the skin in the affected area tender—a mere touch may be almost unendurable. At the same time painful stimuli—for example, the prick of a pin—cause no pain. Other pains of brief duration are deep-seated; they are not so momentary as the superficial ones, and are not attended with cutaneous hyperæsthesia. The second main group of the pains of tabes dorsalis includes those which are prolonged—persisting, though perhaps with varying intensity, for hours, days, or even longer. These are more common in the trunk than the limbs. Most of them are referred to the deeper tissues, though a persisting superficial girdle sensation is not infrequent, and with this there may be a zone of diminished cutaneous sensibility to tactile stimuli. The fixed trunk pains are often unilateral and may be regarded as intercostal neuralgia, as renal colic, or as evidence of hepatic disease. Another possible error is to mistake tabetic pain affecting the sciatic nerve for a simple sciatica. Inquiry for pains in other parts, and the examination of the knee jerks and of the pupils, are the methods which will prevent these errors. Some of these persisting localised deep pains are extremely severe and may produce hours of agony. Another form of fixed pain in tabes consists of widely-diffused sensations, distressing from their nature and persistence rather than from their severity. Tingling, numbness, swelling, feelings of heat and cold, are among the descriptive terms used by patients. The varieties of pain and abnormal sensations met with in tabetics are enormous, and anything like a complete analysis of them would be both cumbersome and useless, but such a general outline as is presented above helps to make observation precise. But apart from pains as part of the general clinical picture of tabes it is necessary to remember that there is one variety of the disease in which pains are the only prominent symptom; the knee-jerk is not lost and there is no ataxia. This condition may certainly persist unchanged for years, so that such cases must be regarded as a special variety of tabes and not merely an early stage of the common or ataxic form; the term "tabetic neuralgia" would appear to be suitable for these cases. The practical importance of this is manifest, as the true nature of the pains may be overlooked and they may be regarded as the result of neuritis, rheumatism, gout, etc. The diagnosis depends upon, first, a recognition of the existence of this special form of tabes, secondly, upon a careful search for other evidences of the disease such, for example, as a defective pupil light response and defective sensibility in the limbs, and thirdly, upon a minute scrutiny of the character of the pains. Deep, dull, prolonged pains may be due to causes other than tabes, but the real "lightning" pains, momentary, stabbing the skin as it were or flashing along it, are hardly ever experienced apart from this disease. The prognosis of these cases, so far as refers to cessation of the pains is not cheerful; on the other hand, such patients are not at all likely to develop ataxia. In the treatment of tabetic pains one has to recognise that though they can often be lessened they can seldom be altogether arrested. It is important to acquaint the patient with the fact that the severity of the pain has no necessary proportion to the activity of the disease. The altered structures are in a condition of instability and are thus susceptible to various disturbing influences, as cold, constipation, and so forth.

Regarding treatment, it is only in the superficial pains that local measures are of value. Chloroform in lint, covered with oiled silk, usually gives temporary relief, but a quarter or a third of a grain of cocaine by hypodermic injection is a more certain proceeding. The electrical method of administering cocaine is worthy of trial in cases of superficial pain and tenderness. The positive electrode of a voltaic battery saturated with a 6 to 10 per cent. solution of cocaine, abolishes the sensibility of the skin both to pain and touch in about 10 minutes, and after a longer application the effect persists for about twice the time the current has been allowed to pass. In deeply-seated pains in the more bulky parts of the limbs, neither cocaine nor any other local measure has much effect, but where the soft tissues are less thick, as in the foot, pains that seem deep and do not cause tenderness, are amenable to an injection of cocaine at the upper part of the region involved. As a rule, these deeper pains can be controlled by internal remedies such as phenacetin, phenazone, and acetanilide. The last-named is, speaking generally, the most effective, though sometimes where this fails one of the others may succeed. Of each it may be said that the best effect is obtained by ordering a single full dose rather than by the frequent repetition of smaller doses, and success is most probable if the remedy is administered at the very onset of the attack of pain. In the most extreme cases of tabetic pain, however, nothing short of morphine proves effective, and large doses may be necessary, but this condition is rare and in no circumstances should morphine be used at the patient's discretion. To lessen the tendency to return of the pain and to reduce both the frequency and severity of the attacks chloride of aluminium in doses of 5 to 10 grs. thrice daily has some value; it is readily soluble in water and is compatible with most other remedies. The salicylate series, for example, sodium salicylate and aspirin, have also some influence in this direction.

